# Platelet Counts in Insoluble Platelet-Rich Fibrin Clots: A Direct Method for Accurate Determination

**DOI:** 10.3389/fbioe.2018.00004

**Published:** 2018-02-01

**Authors:** Yutaka Kitamura, Taisuke Watanabe, Masayuki Nakamura, Kazushige Isobe, Hideo Kawabata, Kohya Uematsu, Kazuhiro Okuda, Koh Nakata, Takaaki Tanaka, Tomoyuki Kawase

**Affiliations:** ^1^Department of Oral and Maxillofacial Surgery, Matsumoto Dental University, Shiojiri, Japan; ^2^Tokyo Plastic Dental Society, Tokyo, Japan; ^3^Implant Dentistry, Nihon University School of Dentistry, Dental Hospital, Tokyo, Japan; ^4^Division of Dental Implantology, Niigata University Medical and Dental Hospital, Niigata, Japan; ^5^Division of Periodontology, Institute of Medicine and Dentistry, Niigata University, Niigata, Japan; ^6^Bioscience Medical Research Center, Niigata University Medical and Dental Hospital, Niigata, Japan; ^7^Department of Materials Science and Technology, Niigata University, Niigata, Japan; ^8^Division of Oral Bioengineering, Institute of Medicine and Dentistry, Niigata University, Niigata, Japan

**Keywords:** platelet-rich fibrin, platelets, tissue-plasminogen activator, white blood cells, clots

## Abstract

Platelet-rich fibrin (PRF) clots have been used in regenerative dentistry most often, with the assumption that growth factor levels are concentrated in proportion to the platelet concentration. Platelet counts in PRF are generally determined indirectly by platelet counting in other liquid fractions. This study shows a method for direct estimation of platelet counts in PRF. To validate this method by determination of the recovery rate, whole-blood samples were obtained with an anticoagulant from healthy donors, and platelet-rich plasma (PRP) fractions were clotted with CaCl_2_ by centrifugation and digested with tissue-plasminogen activator. Platelet counts were estimated before clotting and after digestion using an automatic hemocytometer. The method was then tested on PRF clots. The quality of platelets was examined by scanning electron microscopy and flow cytometry. In PRP-derived fibrin matrices, the recovery rate of platelets and white blood cells was 91.6 and 74.6%, respectively, after 24 h of digestion. In PRF clots associated with small and large red thrombi, platelet counts were 92.6 and 67.2% of the respective total platelet counts. These findings suggest that our direct method is sufficient for estimating the number of platelets trapped in an insoluble fibrin matrix and for determining that platelets are distributed in PRF clots and red thrombi roughly in proportion to their individual volumes. Therefore, we propose this direct digestion method for more accurate estimation of platelet counts in most types of platelet-enriched fibrin matrix.

## Introduction

Owing to their higher growth factor levels, platelet-rich plasma (PRP) and other platelet concentrates have been used for tissue regeneration in a wide range of medical fields, including dentistry (Marx, 2004; Meschi et al., 2016; Panda et al., 2016). Platelet-rich fibrin (PRF) is often designated as second-generation PRP but is distinguished from other PRP derivatives in terms of the elimination of both anticoagulants and coagulation factors (Dohan et al., 2006). Because of its simple preparation protocol, PRF and subsequently developed PRF derivatives have increasingly attracted interest in regenerative dentistry and have been used in clinical settings (Borie et al., 2015; Kawase, 2015; Huang et al., 2017; Miron et al., 2017a,b).

Initially, it was believed that leukocyte- and platelet-rich fibrin does not contain high concentrations of growth factors (Dohan et al., 2006; Gassling et al., 2009), and positive effects of PRF on tissue regeneration were thought to be mainly attributable to the fibrin architecture that effectively functions as a cellular scaffold (Dohan Ehrenfest et al., 2010; Perez et al., 2014). Nevertheless, it was recently demonstrated that PRF and PRF derivatives, e.g., concentrated growth factors (CGF), contain large amounts of growth factors (Lundquist et al., 2008; Su et al., 2009; Kobayashi et al., 2012, 2015; Nishimoto et al., 2015; Takeda et al., 2015; Masuki et al., 2016). We also found that growth factors are present in the fibrin clot exudate, i.e., in serum, and in fibrin fibers (Kobayashi et al., 2012). On the other hand, the characteristics of the presence of growth factors and the number of platelets distributed in these fibrin matrices and other fractions remain poorly understood.

For estimation of platelet counts in PRF and PRF-like clots, a “subtraction method” is often used (Dohan Ehrenfest et al., 2010; Aggarwal and Singhal, 2015; Eren et al., 2016). According to this method, the platelet counts in PRF can be estimated by subtracting those in the clot exudate, in the supernatant serum, and in the red blood cell (RBC) fraction from platelet counts in the initial whole-blood sample. However, this method does not take into account the possibility of a significant number of platelets present in the clotted RBC fraction, i.e., the red thrombus, or the possible loss of (and damage to) platelets during processing for cell counting. In our previous simulation study (Watanabe et al., 2017), we demonstrated that platelets can also be distributed outside the plasma fraction corresponding to fibrin matrices without inducing coagulation. Nevertheless, we could not successfully disperse the platelets aggregated and trapped by fibrin fibers.

After repeated trials and errors, we developed a method that effectively disperses platelets from insoluble fibrin clots with minimal damage and accurately determined platelet counts. In this study, we collected whole-blood samples with or without an anticoagulant to prepare platelet-enriched fibrin clots by CGF-specific centrifugation with or without CaCl_2_, respectively. We validated the proposed method using these kinds of fibrin matrices and tested it on PRF clots. In addition to platelet counts, we attempted to determine white blood cell (WBC) counts because the necessity of WBC inclusion in platelet concentrates has been controversial in the field of regenerative dentistry (Anitua et al., 2015; Kawase, 2015; Choukroun and Ghanaati, 2017). It should be noted that we used the generic term “PRF” here for fibrin matrices prepared from whole-blood samples in this study because the purpose of this study is not to compare genuine PRF with other individual PRF derivatives (Kawase and Tanaka, 2017).

## Materials and Methods

### Preparation of the PRP Fraction and Clotting for Validation

The approved written informed consent documents were presented, and some elements of informed consent were given orally *via* a full and clear but succinct explanation, without jargon or technical terms, to the subjects considering to participation in this project. After obtaining the documents signed by the subjects agreeing to participate, we collected blood samples from four nonsmoking, healthy male subjects with ages ranging from 29 to 57 years. Despite having lifestyle-related diseases and taking medication, these donors had no limitations on the activities of daily living. These donors also declared to be free of HIV, HBV, HCV, or syphilis infections. In addition, a prothrombin test was performed on all the blood samples by means of CoaguChek^®^ XS (Roche, Basel, Switzerland), and all the samples were found to be normal. The blood derivatives and other biological waste were disposed of as medical waste at Niigata University Hospital.

The study design and consent forms for all the procedures were approved by the ethics committee for human subjects of the Niigata University School of Medicine (Niigata, Japan) in accordance with the Helsinki Declaration of 1964 as revised in 2013.

Peripheral blood (~9 mL) was collected into plastic vacuum plain blood collection tubes (Neotube^®^; NIPRO, Osaka, Japan) containing 1 mL of the A-formulation of acid-citrate-dextrose (ACD-A; Terumo, Tokyo, Japan) and was immediately centrifuged at 530 × *g* for 10 min. The upper plasma fraction was collected and transferred to fresh tubes and served as a PRP fraction (Watanabe et al., 2017). Platelet and other blood cell numbers were determined on an automated hematology analyzer (pocH 100iV, Sysmex, Kobe, Japan). Based on the Coulter principle (Groves, 1980), this analyzer provides not only data on cell counts but also size (volume) distribution and characterizes the obtained histograms *via* mean platelet volume (MPV), platelet distribution width (PDW), and platelet–large cell ratio (P-LCR) in terms of platelets.

After addition of three glass beads (GBs) (BZ-5; As-one, Osaka, Japan) and 80 µL of a 10% CaCl_2_ solution, PRP fractions in the plastic tubes were centrifuged on a Medifuge centrifugation system (Silfradent S.r.l., Santa Sofia, Italy) (Corigliano et al., 2010). This instrument automatically changes rotational speed four times as follows: 2,700 rpm (2 min), 2,400 rpm (4 min), 2,700 rpm (4 min), and 3,000 rpm (3 min) (Masuki et al., 2016). The resulting samples of a PRP-derived fibrin matrix (PRP-FM) were digested to release the platelets.

### Preparation of PRF Clots from Whole-Blood Samples for the Test of Applicability

As described elsewhere (Isobe et al., 2017a), 9 mL blood samples was collected into vacuum plain glass tubes and treated with an anticoagulant (Plain BD Vacutainer Tube; Becton, Dickinson and Company, Franklin Lakes, NJ, USA). After the counting of blood cells, the blood samples were mixed with an appropriate volume (~200 μL) of the CaCl_2_ solution and immediately centrifuged by means of the Medifuge centrifugation system (Isobe et al., 2017a,b). After elimination of the RBC fractions, the resulting PRF clots were digested without any modifications.

### Digestion with Tissue-Plasminogen Activator (t-PA)

A commercially available recombinant t-PA, alteplase (GRTPA^®^; Mitsubishi Tanabe Pharma Corp., Osaka, Japan) was dissolved in the enclosed water for injection at a concentration of 6 × 10^5^ IU/mL immediately before use.

Before preparation of PRP-FM clots, PRP preparations were subjected to determination of platelet and WBC counts on the automatic analyzer. PRP preparations (2 mL) were then mixed with 1 mL of the t-PA solution (final concentration 2 × 10^5^ IU/mL) and incubated at 37°C for up to 24 h. At various time points, 100 µL aliquots were taken from 3 mL of the PRF–t-PA digestion mixtures for counting blood cells. Total numbers of released platelets and WBCs were calculated for estimating the recovery rates.

The volumes of PRF clots ranged from approximately 2.0 to 3.5 mL. Irrespective of their volume, 1.0 mL of the t-PA solution was added to the clots to a final concentration of approximately 1.3–2.0 × 10^5^ IU/mL and incubated at 37°C with intermittent gentle agitation. As described in the validation protocol for PRP-FM clots, after 3, 6, and 24 h of incubation, 100 µL aliquots was taken out of 2.5–4.5 mL of the PRF–t-PA mixture for estimation of the platelet counts.

### The Subtraction Method and Simulation Method for Determination of Platelet Counts

For the “subtraction method,” the platelet counts contained in fibrin clots were calculated by subtracting those in the clot exudate, in the supernatant serum, and in the RBC fraction (i.e., the red thrombus) from those in the starting whole-blood sample (Dohan Ehrenfest et al., 2010; Aggarwal and Singhal, 2015; Eren et al., 2016).

To validate this indirect method, we designed a “simulation method.” This method directly determined cell distribution in each fraction in the presence and absence of an anticoagulant (Watanabe et al., 2017).

### Scanning Electron Microscopy (SEM)

Platelets in the liquid PRP fractions or those released from the digested PRF and platelet-like particles were plated on to plastic dishes for 10 min and fixed with 2.5% neutralized glutaraldehyde, serially dehydrated in ethanol and *t*-butanol solutions, and freeze dried. They were then examined under a scanning electron microscope (TM-1000; Hitachi, Tokyo, Japan) with an accelerating voltage of 15 kV, as described previously (Kawase et al., 2014; Isobe et al., 2017b).

### Flow-Cytometric Analysis

The platelet fractions were isolated from whole-blood samples by centrifugation (530 × *g*, 10 min) or from digested fibrin matrices, washed twice with PBS, resuspended in PBS at a density of 1–2 × 10^8^/mL, and fixed with an equal volume of a commercial fixative, ThromboFix (Beckman-Coulter, Brea, CA, USA). After 30-min incubation, platelets were washed twice with PBS and probed with both a fluorescein isothiocyanate-conjugated mouse monoclonal anti-CD41 antibody (BioLegend, San Diego, CA, USA) for 40 min at ambient temperature. After two washes with PBS, platelets were analyzed on a flow cytometer (Cell Lab Quanta SC; Beckman-Coulter Inc., Brea, CA, USA) as described before (Kawabata et al., 2017). For isotype controls, mouse IgG1 (BioLegend) was employed.

### Statistical Analysis

White blood cell and platelet counts represent total number of those cells in fractions and clots unless otherwise specified. The data were expressed as mean ± SD. For multigroup comparisons, statistical analyzes were performed to compare the mean values by one-way analysis of variance, followed by Dunn’s multiple-comparison test (SigmaPlot 12.5; Systat Software, Inc., San Jose, CA, USA). Differences with *P* values of <0.05 were considered statistically significant.

## Results

### Quantification of t-PA Treatment Outcomes

**Figure 1:**
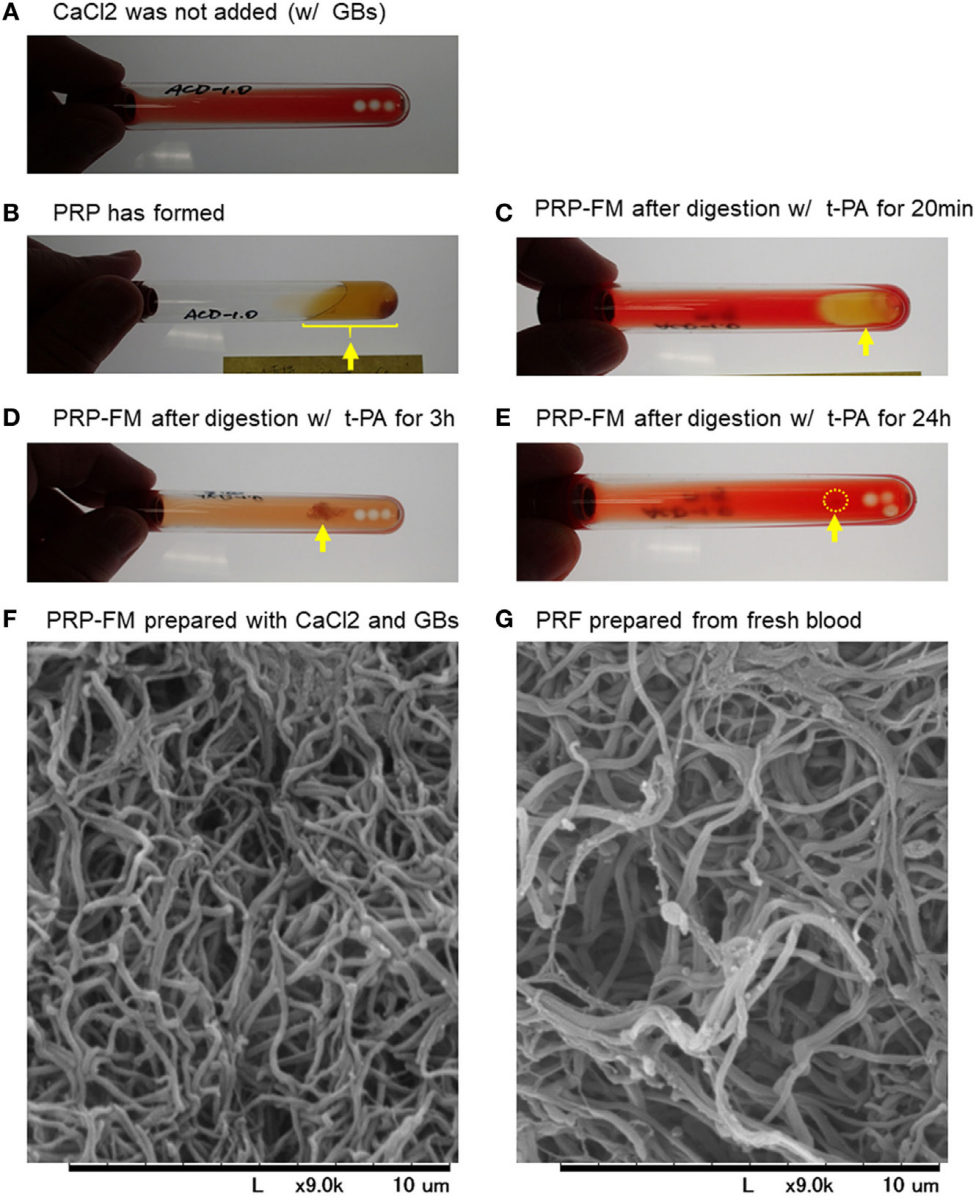
Time-course of degradation images of a platelet-rich plasma (PRP)-FM. The PRP fraction after centrifugation with GBs; CaCl_2_ was not added **(A)**. The PRP fraction after centrifugation with both GBs and added CaCl_2_. The PRP-FM has formed **(B)**. The PRP-FM after digestion with tissue-plasminogen activator (t-PA) for 20 min **(C)**, 3 h **(D)**, and 24 h **(E)**. Abbreviation: GBs, glass beads. Arrows indicate the remaining PRP-FM. Surface microstructures of PRP-FM **(F)** and platelet-rich fibrin (PRF) **(G)**. Similar observations were obtained from other three independent blood samples.

To validate our proposed procedure, we first examined the recovery rate of platelets and WBCs using a PRP-FM. The time-course images of degradation of the PRP-FM are shown in Figure 1. In the plastic tubes, the PRP-FM did not form in the absence of the GBs and/or CaCl_2_ (Figure 1A). By contrast, the combined addition of GBs and CaCl_2_ and centrifugation made it possible for the PRP-FM to form. t-PA potently digested the PRP-FM in a time-dependent manner. After 3 h of digestion, ~70% of the clots were degraded, and this figure increased to over 90% after 24 h (Figures 1B–E). The fibrin fibers that formed in PRP-FM clots were similar to those of PRF clots prepared from whole-blood samples in terms of thickness and cross-link density (Figures 1F,G).

**Figure 2:**
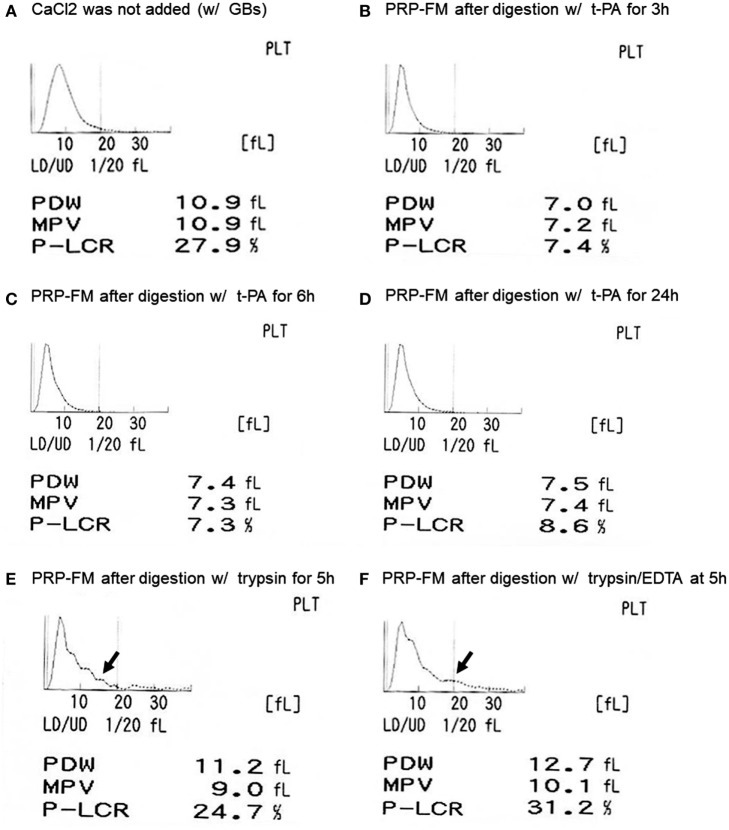
Histograms of platelet size distribution, including genuine platelets and platelet-like particles. Platelet-rich plasma (PRP) fraction after centrifugation with glass beads, without added CaCl_2_ **(A)**. PRP-FM after digestion with tissue-plasminogen activator (t-PA) for 3 h **(B)**, 6 h **(C)**, or 24 h **(D)**. As comparison, PRP-FM after 5 h digestion with trypsin **(E)** or trypsin + EDTA **(F)**. Abbreviations: PDW, platelet distribution width; MPV, mean platelet volume; P-LCR, platelet–large cell ratio.

The size distribution of platelets, including that of the platelet-like particles, is depicted in Figure 2. t-PA treatment reduced P-LCR (Figure 2A vs. Figures 2B–D), which can be viewed in histograms of the size distribution. This sharpened histogram shape persisted for up to 24 h of treatment without further changes (Figures 2B–D). For reference, the distribution histograms of a trypsin-digested PRP-FM in the absence or presence of EDTA are presented in Figures 2E,F. The distribution of larger particles became irregular, and PDW increased slightly as compared with the original plasma fraction.

**Figure 3:**
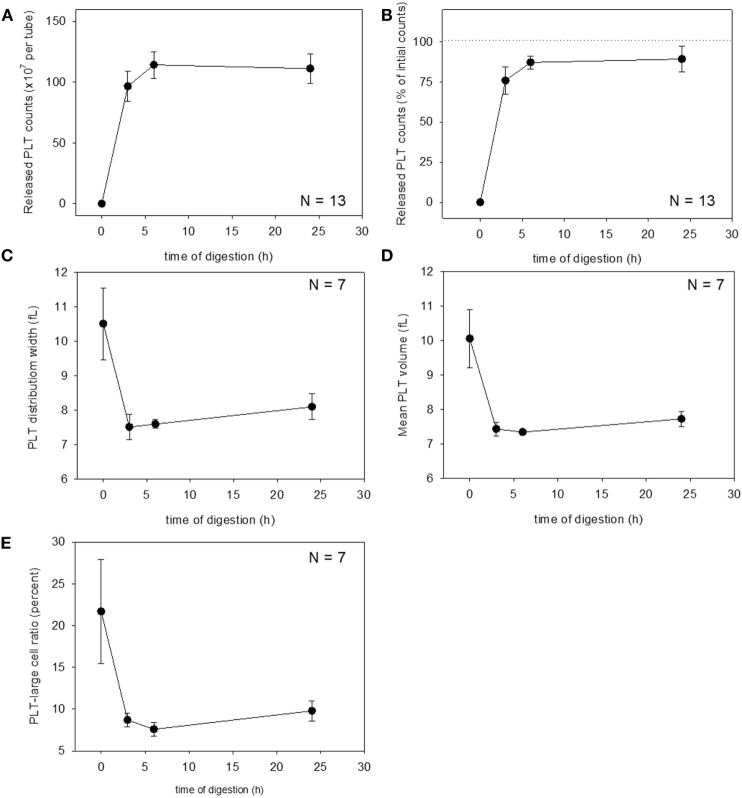
Digestion time–dependent changes in platelet counts and platelet parameters. Total platelet counts released from a platelet-rich plasma-FM as a raw number **(A)** and a percentage **(B)** (*N* = 13). Platelet distribution width **(C)**, mean platelet volume **(D)**, and platelet–large cell ratio **(E)** of the released platelets (*N* = 7).

**Figure 4:**
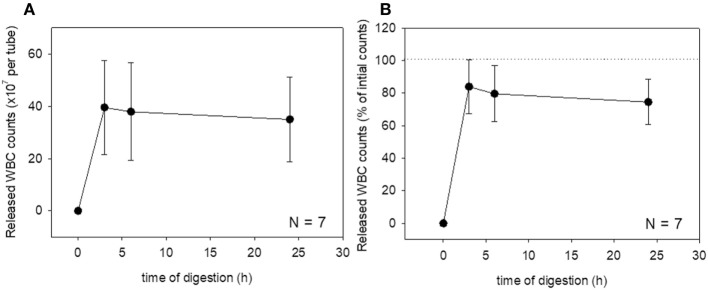
Digestion time-dependent changes in white blood cell (WBC) counts. Total WBC counts released from a platelet-rich plasma-FM as a raw number **(A)** and a percentage **(B)** (*N* = 7).

These changes were then quantified. Changes in platelet counts (both in the estimated number and percentage) and platelet parameters are shown in Figure 3. Approximately 90% of the platelets were released within 6 h of treatment, which was sustained for up to 24 h, although we cannot rule out the possible inclusion of platelet-like particles such as the debris of degraded fibrin and shrunken WBCs. PDW, MPV, and P-LCR were the lowest at 3 and 6 h, and these values slightly increased at 24 h. Changes in WBC counts are shown in Figure 4. In contrast to the platelets, counts of released WBCs peaked within 3 h and declined gradually thereafter.

### Qualitative Validation of the t-PA Treatment

**Figure 5:**
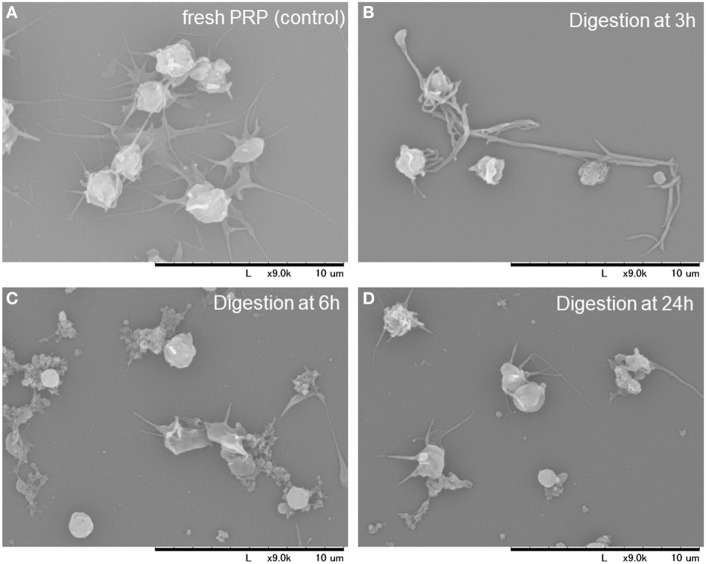
Scanning electron microscopy images of the platelets and other substances present in the digestion mixture at the indicated time points. Platelet-rich plasma (PRP) before clotting **(A)**. PRP-FM digested with tissue-plasminogen activator for 3 h **(B)**, 6 h **(C)**, and 24 h **(D)**.

To rule out the possible contamination with platelet-like particles during the quantitative evaluation described earlier, morphological features of the dispersed particles were examined by SEM. SEM images of platelets and other substances in the digestion mixture at the indicated time points are depicted in Figure 5. During the early phase of treatment (up to 6 h), fibrin fiber fragments were observed, which were further digested into smaller particles after 24 h. Therefore, the digestion mixture after the 24 h treatment seemed to contain only a negligible number of particles that could interfere with the estimation of platelet counts.

### Application of the t-PA Procedure to PRF Digestion

**Figure 6:**
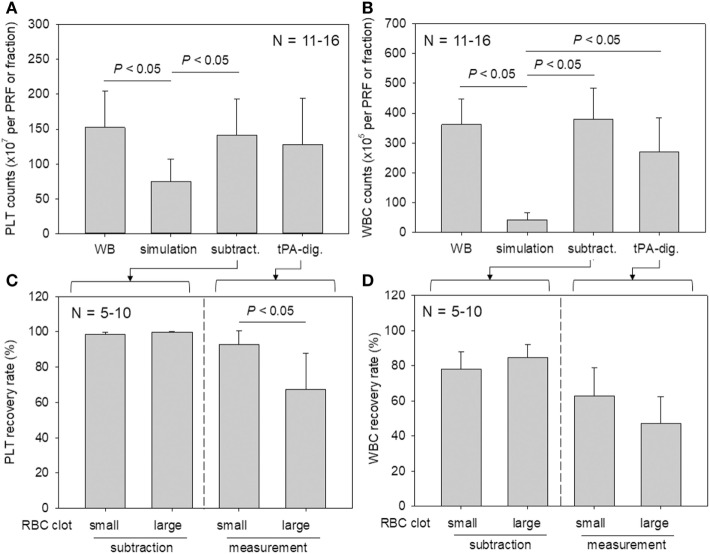
A comparison of platelet and white blood cell (WBC) counting methods (simulation vs. subtraction vs. digestion). **(A,B)** Platelet and WBC counts in platelet-rich fibrin (PRF) clots and the corresponding fractions prepared from whole-blood samples (*N* = 11–6). **(C,D)** The relation between platelet and WBC counts in PRF clots and the sizes of red thrombi (small vs. large) (*N* = 5–10).

Platelet and WBC counts in PRF clots and in the corresponding fractions prepared from whole-blood samples are shown in Figure 6. Our proposed method for determining platelet and WBC counts was compared with the simulation method and subtraction methods. For both counts, the simulation method without formation of fibrin clots appeared to underestimate platelet counts (Watanabe et al., 2017). Our present method yielded data that were comparable to those of the subtraction method.

**Figure 7:**
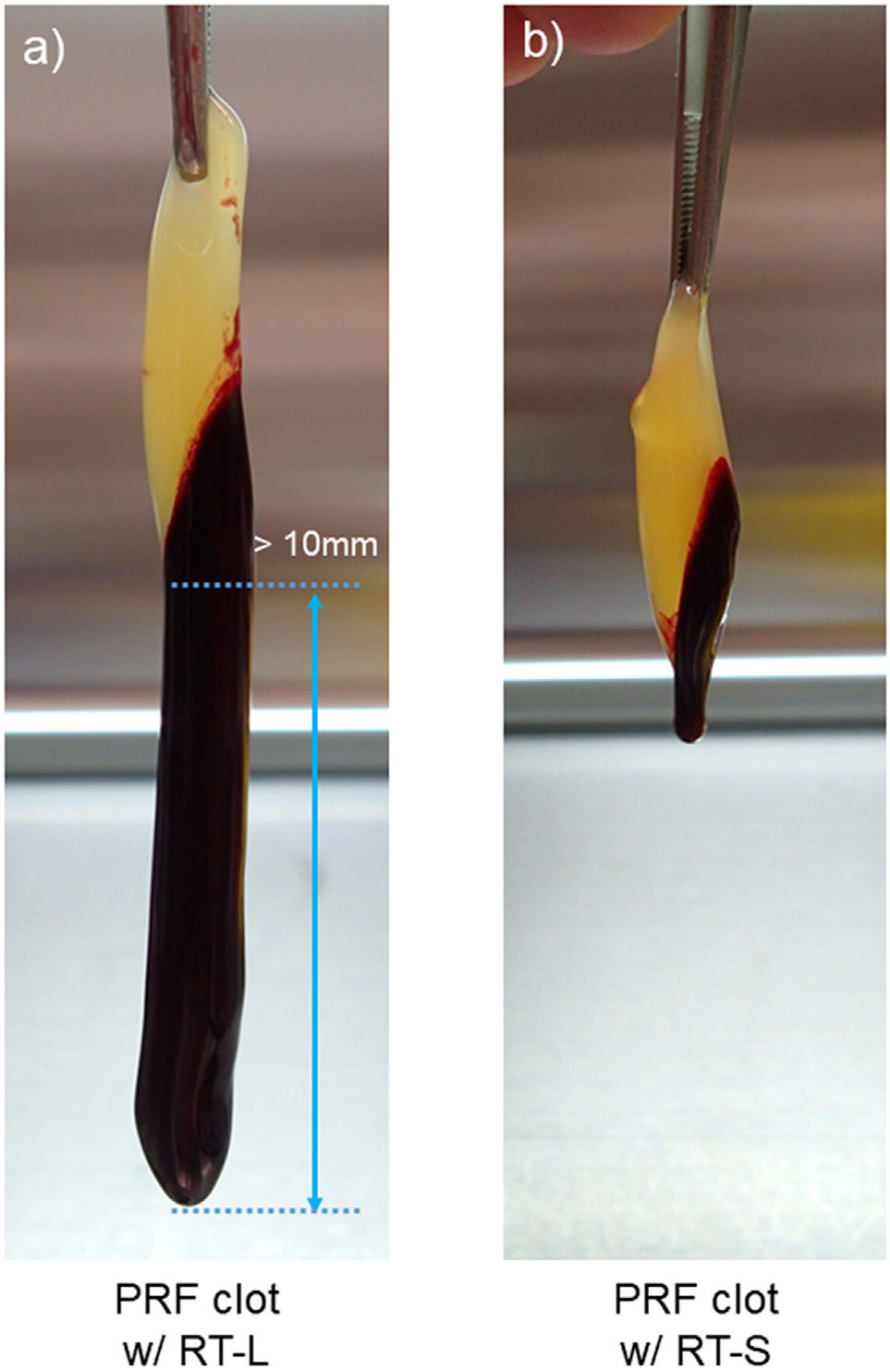
The appearance of clots formed after centrifugation. **(A)** A platelet-rich fibrin (PRF) clot associated with a large red thrombus (>10 mm). **(B)** A PRF clot associated with a small red thrombus (≤10 mm). The average length of PRF clots was 30–40 mm in both cases.

To investigate the platelet distribution in greater detail, we categorized the PRF clots into two groups: clots connected by large red thrombi (>10 mm) and those connected by small red thrombi (≤10 mm; Figure 7); we also determined the platelet and WBC counts. By delaying the centrifugation, we could control the size of red thrombi: when coagulation begins before the centrifugation-dependent fractionation, larger red thrombi are formed. The relations between platelet and WBC counts in the PRF clots and the sizes of red thrombi are presented in Figure 6. In upper panels (Figures 6A,B), platelet and WBC counts in t-PA–digested samples were compared with those of whole-blood samples. Our proposed method revealed that 84.1% of platelets (vs. whole blood) and 74.5% of WBCs (vs. whole blood) were successfully counted in PRF clots. For reference, the subtraction method showed 92.7% of platelets and 101.9% of WBC but underestimated both platelet and WBC counts (49.0 and 11.9%, respectively). These cell counts were further analyzed in two cases (Figures 6C,D): when large red thrombi were tightly associated with the PRF clots, the platelet recovery rate in the PRF clots was significantly lower than that associated with the small red thrombi (67.2 vs. 92.5%). A similar phenomenon was observed in WBC counts (47.1 vs. 62.9%). By contrast, the subtraction method provided similar data on both platelets and WBCs regardless of red thrombus types.

**Figure 8:**
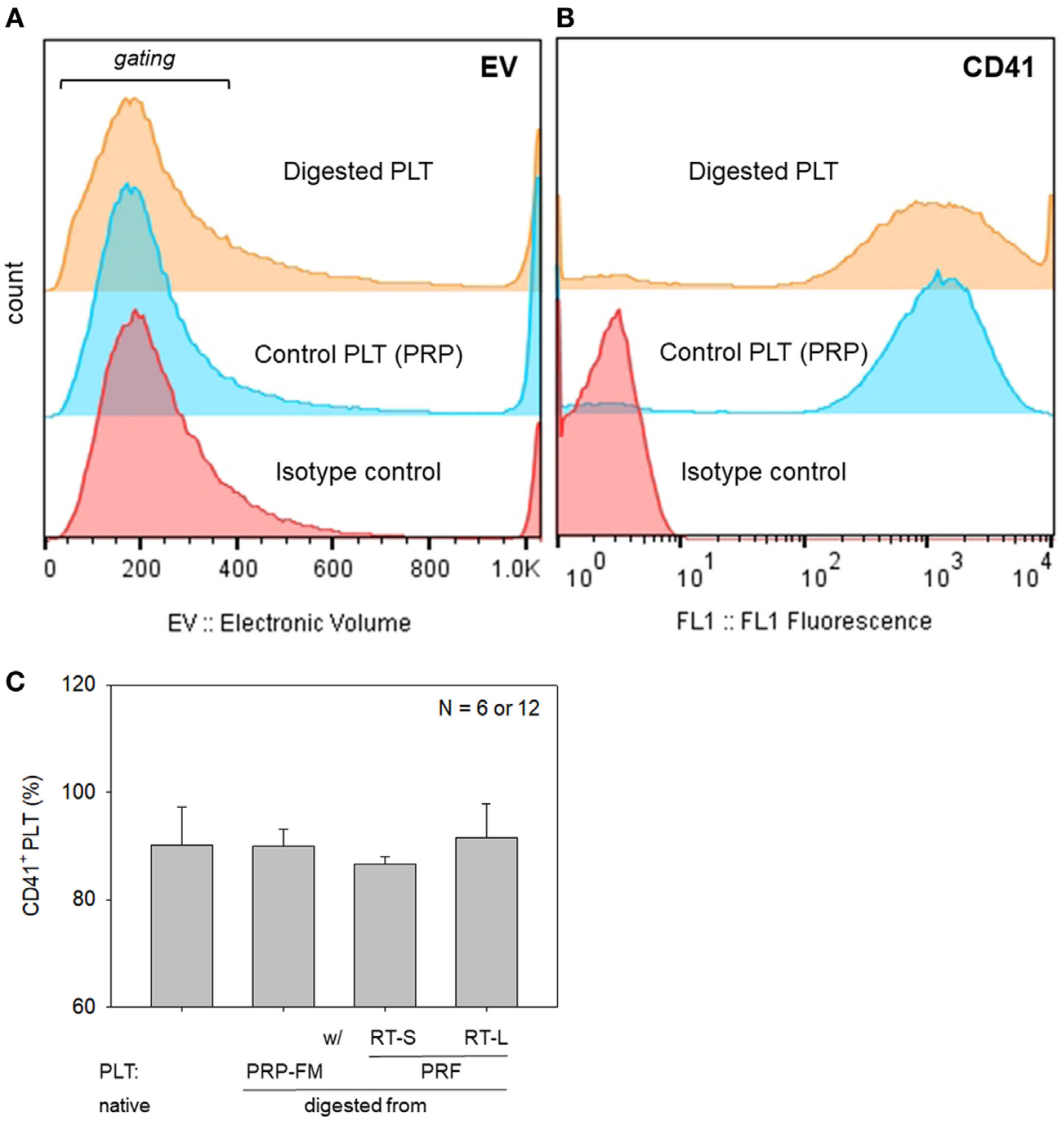
Histograms of particle size, which is expressed as electronic volume (EV), in all populations **(A)**, and CD41^+^ platelets in the gated populations **(B)**. **(C)** The percentage of CD41^+^ platelets in the gated populations. Platelets derived from a platelet-rich plasma (PRP)-FM and whole-blood-derived platelet-rich fibrin (PRF) clots; large and small red thrombi are compared with platelets contained in the PRP fraction before clotting (*N* = 6 or 12).

To further rule out the significant contamination with platelet-like particles, flow-cytometric analysis was carried out with a specific antibody against CD41, which is a convenient marker of platelets (Bagamery et al., 2005). The percentages of CD41^+^ platelets in the populations corresponding to platelets are shown in Figure 8. There were no significant differences in the electronic volume (EV) histogram between the digested and control platelets. Furthermore, there were no significant differences in the percentages of CD41^+^ cells in the gated populations among the samples tested; however, the shapes of the peaks (the range of CD41 expression per individual platelet) were different among the samples.

## Discussion

The platelet count is a quantity but has been accepted as one of the major indexes for ensuring the quality of platelet concentrates (Marx, 2001). Nevertheless, there has been a lack of a method for accurate determination of platelet counts in gel types of platelet concentrates such as PRF. So far, platelet counts have been calculated but not directly determined by the subtraction method. The major drawbacks of this method are discussed in detail in the following paragraph. In summary, however, this method provides data that reflect the sum of errors deriving from operators’ technical skills, surface properties of devices, and the complicated nature of the procedure. Therefore, we have long doubted the accuracy of this indirect method. To improve this state of affairs, in this study, we attempted to develop a direct method, validated it on PRP-FM clots whose platelets are counted before clotting, and applied it to PRF clots. Although WBCs were not successfully recovered by t-PA digestion as much as expected, platelets could be recovered at or beyond the acceptable levels.

Because blood cells are deformable and not ideally spherical, and because their specific gravity depends on individual cell types, fractionation of blood cells varies among the individual blood samples and among centrifugation conditions. For determination of platelet counts in PRF and PRF derivatives, it has generally been accepted that the subtraction method—subtracting platelet counts in the clot exudate, in the supernatant serum, and in the RBC fraction from those in the initial whole-blood sample—is sufficient for estimating the platelet counts in the PRF clot (Dohan Ehrenfest et al., 2010; Aggarwal and Singhal, 2015; Eren et al., 2016). Nonetheless, the pitfall of this complicated indirect method is that platelet counts in whole-blood samples are determined using parallel samples containing an anticoagulant, not the anticoagulant-free samples employed for PRF preparation. In addition, even though blood cells are counted immediately after collection, this indirect method does not take into account a possible platelet loss during the preparation and estimation processes. For example, platelets can easily adhere to the inside wall of glass tubes with insufficient silicon coating, to uncoated stainless-steel compression devices, and to dry gauze (Kobayashi et al., 2012; Watanabe et al., 2017), or may be damaged during squeezing of exudates from clots by strong compression (Dohan Ehrenfest et al., 2010). In addition, significant numbers of aggregated platelets are a part of red thrombi (Watanabe et al., 2017) and therefore cannot be counted by the subtraction method. Thus, we have emphasized the necessity of a direct method for determining platelet counts to investigate the possible relation between the bioactivity and platelet counts in PRF and PRF derivatives in more detail. Comparative evaluation of the conventional subtraction method and the proposed digestion method is summarized in Table 1. In brief, compared with the subtraction method, the major advantages of our proposed method are (1) high accuracy, (2) simple procedure, (3) no technical skill, and (4) no limitation to types of fibrin matrix, while the major disadvantages are (1) long completion time, (2) cost of the reagent (t-PA), (3) requirement of incubator, and (4) additional tubes required for growth factor assays.

**Table 1:** Comparison between the conventional subtraction method and the proposed digestion method.

	Subtraction method	Digestion method
Accuracy	Medium	High
Approach	Indirect	Direct
Procedure	Complicated	Simple
Time for completion	Medium	Long
Operators’ skill	High	Low
Error range	Wide	Narrow
Cost	Low	Medium
Specific instruments	None (or compression device)	Incubator
Correlation with growth factor levels	Possible in single tubes	Parallel tubes of samples required
Applicability	Limited to platelet-rich fibrin and its derivatives (the original whole-blood samples and the resulting liquid fractions required)	No limitation

In a preliminary study, we tested several proteases, such as trypsin, dispase, and plasmin; however, these reagents appeared to poorly disperse platelets or seriously damage platelets, judging by the data on a platelet size distribution and SEM observations. t-PA significantly reduced the size of platelets, but did not produce significant amounts of platelet-like debris, i.e., fibrin fragments. Therefore, these findings suggested that platelets can be effectively dispersed by t-PA from fibrin matrices containing trapped platelets with minimal damage to (or loss of) platelets or overestimation of platelet counts. We validated the accuracy of this digestion method on PRP-FM and subsequently tested the method on PRF clots in this study. On the other hand, we believe that this method can be applied to most types of fibrin matrices where platelets are trapped, including advanced-PRF and CGF, because their mechanical and degradation properties and microstructure of the fibrin fiber meshwork are almost identical to one another (Isobe et al., 2017b).

As for the difference between t-PA and plasmin, several possible points can be considered and discussed. It is known that t-PA, a serine protease that converts the proenzyme plasminogen to the proteinase plasmin, is specifically bound to fibrin along with plasminogen, and that t-PA activity can be enhanced over 100-fold through specific binding (Nieuwenhuizen, 2001; Kruithof and Dunoyer-Geindre, 2014). Accordingly, it can be predicted that the plasmin converted by t-PA on fibrin fibers is capable of degrading fibrin fibers effectively. By contrast, it is likely that exogenously added plasmin binds to fibrin less effectively, and consequently degrades fibrin fibers with lower potency than on-site converted plasmin doses. Furthermore, endogenous α2-antiplasmin, if present in the clots, may interfere with the action of exogenous plasmin, which cannot be ignored (Palta et al., 2014).

**Figure 9:**
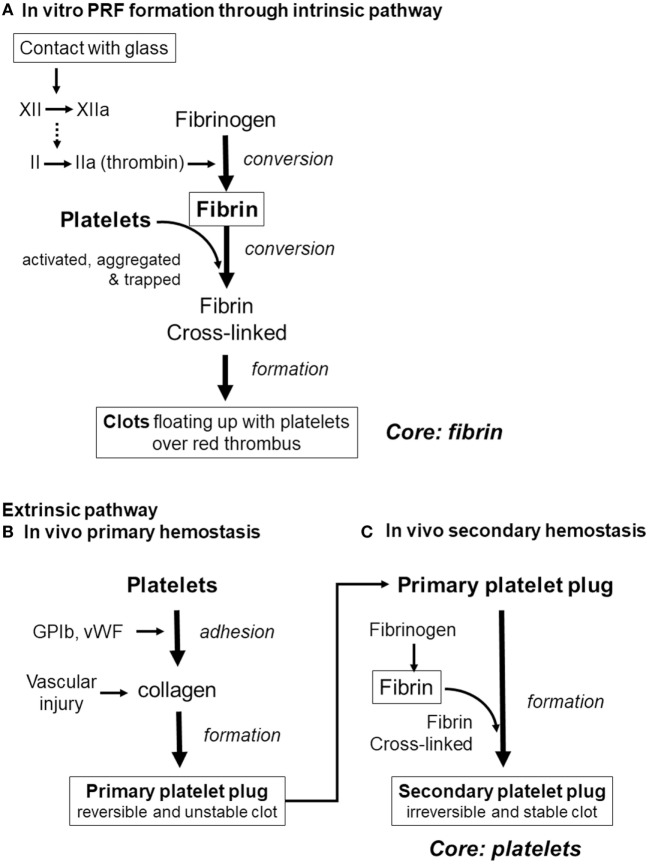
Differences in the pathways of clot formation. **(A)** *In vitro* platelet-rich fibrin (PRF) cloy formation. **(B)** *In vivo* primary hemostasis of the extrinsic pathway. **(C)** *In vivo* secondary hemostasis of the extrinsic pathway.

Finally, we would like to discuss the major possible pathways of clot formation by comparing fibrin clot formation *in vitro* (which results from activation of the intrinsic coagulation pathway) with *in vivo* extrinsic hemostasis (Figure 9). *In vivo* platelet activation and aggregation precede clot formation. Therefore, it is possible that platelet aggregates form the core of the secondary platelet plugs, i.e., blood clots (Palta et al., 2014), and it is widely accepted that platelets cannot be easily dispersed from blood clots. By contrast, during *in vitro* PRF formation, activation of factor XII in the coagulation cascade (Vogler and Siedlecki, 2009), consequently inducing conversion of fibrinogen to fibrin, precedes platelet activation, aggregation, and adhesion to fibrin fibers. In support of this mechanism, we previously reported that platelet aggregates are located predominantly on the surface of a clot, and at the interface between upper conventional PRF and the lower red thrombus (Kobayashi et al., 2012). Thus, we can theorize that it is easier to release platelets by fibrin digestion from PRF clots.

## Conclusion

Here, we demonstrated that t-PA is potent enough to successfully disperse platelets aggregated in insoluble fibrin matrices enriched with platelets. We believe that this methodology will be helpful in detailed studies on the relation between growth factor levels and platelet counts for any types of platelet concentrates.

## Ethics Statement

The approved written informed consent documents were presented, and some elements of informed consent were given orally *via* a full and clear but succinct explanation, without jargon or technical terms, to the subjects considering to participation in this project. After obtaining the documents signed by the subjects agreeing to participate, we collected blood samples from four nonsmoking, healthy male subjects with ages ranging from 29 to 57 years. Despite having lifestyle-related diseases and taking medication, these donors had no limitations on the activities of daily living. These donors also declared to be free of HIV, HBV, HCV, or syphilis infections. In addition, a prothrombin test was performed on all the blood samples by means of CoaguChek^®^ XS (Roche, Basel, Switzerland), and all the samples were found to be normal. The blood derivatives and other biological waste were disposed of as medical waste at Niigata University Hospital. The study design and consent forms for all the procedures were approved by the ethics committee for human subjects of the Niigata University School of Medicine (Niigata, Japan) in accordance with the Helsinki Declaration of 1964 as revised in 2013.

## Author Contributions

YK, TT, and TK conceived and designed the study. YK, TW, and TK performed the experiments and wrote the manuscript. MN, KI, HK, KU, and KO performed the experiments and data analysis. KN participated in data interpretation and manuscript preparation. All the authors read and approved the final version of the manuscript.

## Conflict of Interest Statement

The authors declare that the research was conducted in the absence of any commercial or financial relationships that could be construed as a potential conflict of interest.

## References

[B1] Aggarwal A. Singhal N. (2015). Evaluation of content and distribution of platelets in platelet rich fibrin at various centrifugation time periods: a light microscopic study. Int. J. Dent. Med. Res. 1, 61–64.

[B2] Anitua E. Zalduendo M. Troya M. Padilla S. Orive G. (2015). Leukocyte inclusion within a platelet rich plasma-derived fibrin scaffold stimulates a more pro-inflammatory environment and alters fibrin properties. PLoS ONE 10:e0121713. 10.1371/journal.pone.0121713 25823008 PMC4379078

[B3] Bagamery K. Kvell K. Landau R. Graham J. (2005). Flow cytometric analysis of CD41-labeled platelets isolated by the rapid, one-step OptiPrep method from human blood. Cytometry A 65, 84–87. 10.1002/cyto.a.20133 15779060

[B4] Borie E. Olivi D. G. Orsi I. A. Garlet K. Weber B. Beltran V. (2015). Platelet-rich fibrin application in dentistry: a literature review. Int. J. Clin. Exp. Med. 8, 7922–7929. 26221349 PMC4509294

[B5] Choukroun J. Ghanaati S. (2017). Reduction of relative centrifugation force within injectable platelet-rich-fibrin (PRF) concentrates advances patients’ own inflammatory cells, platelets and growth factors: the first introduction to the low speed centrifugation concept. Eur. J. Trauma Emerg. Surg. 10.1007/s00068-017-0767-9 28283682 PMC5808086

[B6] Corigliano M. Sacco L. Baldoni E. (2010). CGF-una proposta terapeutica per la medicina rigenerativa. Odontoiatria 1, 69–81.

[B7] Dohan D. M. Choukroun J. Diss A. Dohan S. L. Dohan A. J. Mouhyi J. (2006). Platelet-rich fibrin (PRF): a second-generation platelet concentrate. Part I: technological concepts and evolution. Oral Surg. Oral Med. Oral Pathol. Oral Radiol. Endod. 101, e37–e44. 10.1016/j.tripleo.2005.07.008 16504849

[B8] Dohan Ehrenfest D. M. Del Corso M. Diss A. Mouhyi J. Charrier J. B. (2010). Three-dimensional architecture and cell composition of a Choukroun’s platelet-rich fibrin clot and membrane. J. Periodontol. 81, 546–555. 10.1902/jop.2009.090531 20373539

[B9] Eren G. Gurkan A. Atmaca H. Donmez A. Atilla G. (2016). Effect of centrifugation time on growth factor and MMP release of an experimental platelet-rich fibrin-type product. Platelets 27, 427–432. 10.3109/09537104.2015.1131253 26830681

[B10] Gassling V. L. Acil Y. Springer I. N. Hubert N. Wiltfang J. (2009). Platelet-rich plasma and platelet-rich fibrin in human cell culture. Oral Surg. Oral Med. Oral Pathol. Oral Radiol. Endod. 108, 48–55. 10.1016/j.tripleo.2009.02.007 19451011

[B11] Groves M. R. (1980). Application of the electrical sizing principle of Coulter to a new multiparameter system. IEEE Trans. Biomed. Eng. 27, 364–369. 10.1109/tbme.1980.326649 7409801

[B12] Huang Y. Bornstein M. M. Lambrichts I. Yu H. Y. Politis C. Jacobs R. (2017). Platelet-rich plasma for regeneration of neural feedback pathways around dental implants: a concise review and outlook on future possibilities. Int. J. Oral Sci. 9, 1–9. 10.1038/ijos.2017.1 28282030 PMC5379164

[B13] Isobe K. Suzuki M. Watanabe T. Kitamura Y. Suzuki T. Kawabata H. (2017a). Platelet-rich fibrin prepared from stored whole-blood samples. Int. J. Implant. Dent. 3, 6 10.1186/s40729-017-0068-4 28251561 PMC5332319

[B14] Isobe K. Watanebe T. Kawabata H. Kitamura Y. Okudera T. Okudera H. (2017b). Mechanical and degradation properties of advanced platelet-rich fibrin (A-PRF), concentrated growth factors (CGF), and platelet-poor plasma-derived fibrin (PPTF). Int. J. Implant. Dent. 3, 17 10.1186/s40729-017-0081-7 28466249 PMC5413460

[B15] Kawabata H. Isobe K. Watanabe T. Okudera T. Nakamura M. Suzuki M. (2017). Quality assessment of platelet-rich fibrin-like matrix prepared from whole blood samples after extended storage. Biomedicines 5, 57 10.3390/biomedicines5030057 PMC5618315 28926988

[B16] Kawase T. (2015). Platelet-rich plasma and its derivatives as promising bioactive materials for regenerative medicine: basic principles and concepts underlying recent advances. Odontology 103, 126–135. 10.1007/s10266-015-0209-2 26040505

[B17] Kawase T. Kamiya M. Kobayashi M. Tanaka T. Okuda K. Wolff L. F. (2014). The heat-compression technique for the conversion of platelet-rich fibrin preparation to a barrier membrane with a reduced rate of biodegradation. J. Biomed. Mater. Res. Part B Appl. Biomater. 103, 825–831. 10.1002/jbm.b.33262 25132655

[B18] Kawase T. Tanaka T. (2017). An updated proposal for terminology and classification of platelet-rich fibrin. Renerative Therapy 7, 80–81. 10.1016/j.reth.2017.10.002 PMC6153447 30271855

[B19] Kobayashi M. Kawase T. Horimizu M. Okuda K. Wolff L. F. Yoshie H. (2012). A proposed protocol for the standardized preparation of PRF membranes for clinical use. Biologicals 40, 323–329. 10.1016/j.biologicals.2012.07.004 22841724

[B20] Kobayashi M. Kawase T. Okuda K. Wolff L. F. Yoshie H. (2015). In vitro immunological and biological evaluations of the angiogenic potential of platelet-rich fibrin preparations: a standardized comparison with PRP preparations. Int. J. Implant. Dent. 1, 31. 10.1186/s40729-015-0032-0 27747653 PMC5005601

[B21] Kruithof E. K. Dunoyer-Geindre S. (2014). Human tissue-type plasminogen activator. Thromb. Haemost. 112, 243–254. 10.1160/th13-06-0517 24718307

[B22] Lundquist R. Dziegiel M. H. Agren M. S. (2008). Bioactivity and stability of endogenous fibrogenic factors in platelet-rich fibrin. Wound Repair Regen. 16, 356–363. 10.1111/j.1524-475X.2007.00344.x 18282265

[B23] Marx R. E. (2001). Platelet-rich plasma (PRP): what is PRP and what is not PRP? Implant Dent. 10, 225–228. 10.1097/00008505-200110000-00002 11813662

[B24] Marx R. E. (2004). Platelet-rich plasma: evidence to support its use. J. Oral Maxillofac. Surg. 62, 489–496. 10.1016/j.joms.2003.12.003 15085519

[B25] Masuki H. Okudera T. Watanabe T. Suzuki M. Nishiyama K. Okudera H. (2016). Growth factor and pro-inflammatory cytokine contents in PRP, plasma rich in growth factors (PRGF), advanced-platelet-rich fibrin (A-PRF) and concentrated growth factors (CGF). Int. J. Implant. Dent. 2, 19 10.1186/s40729-016-0052-4 27747711 PMC5005757

[B26] Meschi N. Castro A. B. Vandamme K. Quirynen M. Lambrechts P. (2016). The impact of autologous platelet concentrates on endodontic healing: a systematic review. Platelets 27, 613–633. 10.1080/09537104.2016.1226497 27658056

[B27] Miron R. J. Bishara M. Choukroun J. (2017a). Basics of platelet-rich fibrin therapy. Dent. Today 36, 74–76. 29235312

[B28] Miron R. J. Fujioka-Kobayashi M. Bishara M. Zhang Y. Hernandez M. Choukroun J. (2017b). Platelet-rich fibrin and soft tissue wound healing: a systematic review. Tissue Eng. Part B Rev. 23, 83–99. 10.1089/ten.TEB.2016.0233 27672729

[B29] Nieuwenhuizen W. (2001). Fibrin-mediated plasminogen activation. Ann. N. Y. Acad. Sci. 936, 237–246. 10.1111/j.1749-6632.2001.tb03512.x 11460481

[B30] Nishimoto S. Fujita K. Sotsuka Y. Kinoshita M. Fujiwara T. Kawai K. (2015). Growth factor measurement and histological analysis in platelet rich fibrin: a pilot study. J. Maxillofac. Oral Surg. 14, 907–913. 10.1007/s12663-015-0768-3 26604462 PMC4648775

[B31] Palta S. Saroa R. Palta A. (2014). Overview of the coagulation system. Indian J. Anaesth. 58, 515–523. 10.4103/0019-5049.144643 25535411 PMC4260295

[B32] Panda S. Doraiswamy J. Malaiappan S. Varghese S. S. Del Fabbro M. (2016). Additive effect of autologous platelet concentrates in treatment of intrabony defects: a systematic review and meta-analysis. J. Investig. Clin. Dent. 7, 13–26. 10.1111/jicd.12117 25048153

[B33] Perez A. G. Rodrigues A. A. Luzo A. C. Lana J. F. Belangero W. D. Santana M. H. (2014). Fibrin network architectures in pure platelet-rich plasma as characterized by fiber radius and correlated with clotting time. J. Mater. Sci. Mater. Med. 25, 1967–1977. 10.1007/s10856-014-5235-z 24838297

[B34] Su C. Y. Kuo Y. P. Tseng Y. H. Su C. H. Burnouf T. (2009). In vitro release of growth factors from platelet-rich fibrin (PRF): a proposal to optimize the clinical applications of PRF. Oral Surg. Oral Med. Oral Pathol. Oral Radiol. Endod. 108, 56–61. 10.1016/j.tripleo.2009.02.004 19451002

[B35] Takeda Y. Katsutoshi K. Matsuzaka K. Inoue T. (2015). The effect of concentrated growth factor on rat bone marrow cells in vitro and on calvarial bone healing in vivo. Int. J. Oral Maxillofac. Implants 30, 1187–1196. 10.11607/jomi.3995 26394358

[B36] Vogler E. A. Siedlecki C. A. (2009). Contact activation of blood plasma coagulation: a contribution from the hematology at Biomaterial Interfaces Research Group the Pennsylvania State University. Biomaterials 30, 1857–1869. 10.1016/j.biomaterials.2008.12.041 19168215 PMC2705825

[B37] Watanabe T. Isobe K. Suzuki T. Kawabata H. Nakamura M. Tsukioka T. (2017). An evaluation of the accuracy of the subtraction method used for determining platelet counts in advanced platelet-rich fibrin and concentrated growth factor preparations. Dent. J. 5, 7 10.3390/dj5010007 PMC5806990 29563413

